# Adaptive evolution of the symbiotic gene *NORK *is not correlated with shifts of rhizobial specificity in the genus *Medicago*

**DOI:** 10.1186/1471-2148-7-210

**Published:** 2007-11-06

**Authors:** Stéphane De Mita, Sylvain Santoni, Joëlle Ronfort, Thomas Bataillon

**Affiliations:** 1 UMR 1097 Diversité et Adaptation des Plantes Cultivées – INRA Montpellier, France; 2 Bioinformatic Research Center – Institute of Biology, Section of Genetics and Ecology, University of Aarhus, Aarhus, Denmark; 3 Laboratory of Molecular Biology, Wageningen University, P.O. Box 8128, 6700 ET Wageningen, The Netherlands

## Abstract

**Background:**

The *NODULATION RECEPTOR KINASE *(*NORK*) gene encodes a Leucine-Rich Repeat (LRR)-containing receptor-like protein and controls the infection by symbiotic rhizobia and endomycorrhizal fungi in Legumes. The occurrence of numerous amino acid changes driven by directional selection has been reported in this gene, using a limited number of messenger RNA sequences, but the functional reason of these changes remains obscure. The *Medicago *genus, where changes in rhizobial associations have been previously examined, is a good model to test whether the evolution of *NORK *is influenced by rhizobial interactions.

**Results:**

We sequenced a region of 3610 nucleotides (encoding a 392 amino acid-long region of the NORK protein) in 32 *Medicago *species. We confirm that positive selection in *NORK *has occurred within the *Medicago *genus and find that the amino acid positions targeted by selection occur in sites outside of solvent-exposed regions in LRRs, and other sites in the N-terminal region of the protein. We tested if branches of the *Medicago *phylogeny where changes of rhizobial symbionts occurred displayed accelerated rates of amino acid substitutions. Only one branch out of five tested, leading to *M. noeana*, displays such a pattern. Among other branches, the most likely for having undergone positive selection is not associated with documented shift of rhizobial specificity.

**Conclusion:**

Adaptive changes in the sequence of the NORK receptor have involved the LRRs, but targeted different sites than in most previous studies of LRR proteins evolution. The fact that positive selection in *NORK *tends not to be associated to changes in rhizobial specificity indicates that this gene was probably not involved in evolving rhizobial preferences. Other explanations (*e.g*. coevolutionary arms race) must be tested to explain the adaptive evolution of *NORK*.

## Background

Many plants allow intracellular accommodation of symbiotic microorganisms in order to improve nutrient uptake. The most ubiquitous example is vesicular arbuscular mycorrhizae (VAM) which are specific organs contributed by plant roots and fungi [1]. VAM are probably as old as the common ancestor of all land plants [2] and most of them are described as generalist (*i.e*. many distantly related fungi can associate with a given plant). In contrast, only plants of the Fabaceae family (legumes), plus the Ulmaceae *Parasponia*, harbor nitrogen-fixing bacteria in specific root organs (nodules). A polyphyletic group of symbiotic bacteria collectively named rhizobia are able to trigger nodule organogenesis [3]. The legume-rhizobium symbiosis is characterized by host-symbiont specificity controlled by stringent partner recognition. As a result, only a limited range of bacteria can nodulate a given legume species. Despite their differences in levels of specificity, VAM and rhizobial symbioses exhibit the same identical host cellular responses at early stages of the interaction. It is possible that VAM have served as a pre-adaptation to the rhizobial symbiosis [4]. The specificity in legume-rhizobium symbiosis is mediated by signaling molecules released by both partners, among which rhizobial Nod factors are thought to be the most important [5]. Recently, VAM fungi have also been shown to release signaling molecules as well [6]. There is mounting evidence that the same genes are activated during the establishment of both rhizobial and fungal symbioses [7].

The gene *NORK *(*NODULATION RECEPTOR KINASE*) of *Medicago truncatula *[8] controls a common signaling pathway required for some of the earliest stages of Nod factor signaling, but also mycorrhizal signaling. *NORK *encodes a transmembrane protein with structural analogy to receptor kinases involved in signaling or disease resistance. Its extracellular region contains a large N-terminal which has not yet been functionally characterized. The C-terminal region of the extracellular domain (closer to the transmembrane domain in the predicted primary structure) contains three Leucine-Rich Repeats (LRRs) which are motifs involved in ligand binding. LRRs appear in many proteins involved in disease resistance or cellular signaling. LRRs are expected to conform as aligned β sheets in which solvent-exposed (non-Leucine) residues control ligand specificity [9].

Current hypotheses regarding its functional role propose that NORK is involved in a complex Nod factor receptor [10] or that it plays an intermediary part and connects the activity of Nod factor (and mycorrhizal) receptors to subsequent steps [11]. However, no functional experiments testing these hypotheses have been reported yet. We previously examined patterns of within- and between-species nucleotide variation in *NORK *and found footprints of positive selection in the phylogeny of six legume species from five different genera but no evidence of positive selection in the polymorphism of *Medicago truncatula *[12]. The finding of positive selection was based on messenger RNA (mRNA) sequences spanning the entire coding region of the gene but available for only a few species. Signatures of positive selection were clearly located in the region encoding the LRRs. Even if *NORK *is a key component of the Nod factor signaling pathway, it is not obvious that the legume-rhizobium symbiosis is the only explanation for the selective pressures found in that gene. We aimed to investigate whether *NORK *evolution can be linked specifically to the evolution of rhizobial symbiosis, focusing on shifts in host specificity.

The *Medicago *genus is the only available fine-scale case study of the patterns of rhizobia specificity [13]. Most *Medicago *species are associated to two sister species, *Sinorhizobium medicae *and *S. meliloti*. However, basal species are associated to more specific, Near-East strains (making the identification of the ancestral state impossible). Two non-basal *Medicago *species (*M. noeana *and *M. rigiduloides*) seem to have reverted to this preference for Near-East strains. Two sister species *M. laciniata *and *M. sauvagei *are associated to the specific group of *S. meliloti *bv. medicaginis [14], and *M. monspeliaca *is associated to another group. This pattern suggests several important transitions of symbiotic preference along the *Medicago *phylogeny.

Here we present a detailed analysis of the evolution of *NORK *using an intensive sampling of species from the genus *Medicago *and focusing on the LRR region. Species included in our sample were selected to encompass evolutionary transitions that occurred during the evolution of the genus *Medicago*. We examined a region of 3610 nucleotides coding for the extracellular domain of NORK. We sequenced 32 *Medicago *species and used 6 mRNA sequences from other genera. A widely used indicator of selective constraint is the ratio of non-synonymous to synonymous substitution rates [15]. This parameter is referred to as *ω *and can be estimated from aligned coding sequence data using a maximum-likelihood method [16] which allows testing various hypotheses. The data exhibit strong evidence that *NORK *has undergone positive selection in the history of the *Medicago *genus. We present an estimation of the proportion of sites under selection and their position in the gene. We test whether branches of the *Medicago *phylogeny which are associated to changes in rhizobial preferences exhibit evidence of positive selection. We find that there is no obvious such influence whereas at least one branch associated to no known transition in symbiotic specificity has almost certainly undergone positive selection.

## Results

### Sequence data

A region of 3610 nucleotides was sequenced in 32 *Medicago *species (pairwise identity: minimum 0.908, maximum 0.996, mean 0.942). Positions of exons were determined by homology with *M. truncatula*. A protein-coding region of 1178 nucleotides was extracted and aligned with mRNA sequences of 6 species from other genera (pairwise identity: minimum 0.811, maximum 0.996, mean 0.932). This alignment was translated in an alignment of 392 amino acids (pairwise identity: minimum 0.730, maximum 0.997, mean 0.900). The NORK protein has undergone a few insertion and deletion events. They are restricted to a short track of the extracellular domain (not in the LRRs). Within *Medicago *(and using mRNA sequences of *Lotus *and *Sesbania *as outgroups) we observe two short deletions (positions 200 and 221, numbered with respect to the *M. truncatula *protein sequence CAD10809) and one short insertion (two residues between 240 and 241). Outside *Medicago *there are only two additional short insertion/deletion events: a two-residue insertion at positions 214–215 common to all *Medicago *and a single amino acid insertion/deletion at position 373 (the only insertion occurring outside the 200–241 range). Hence NORK has undergone relatively few rearrangement events within the Hologalegina clade [17].

### Significant excess of non-synonymous substitutions in *NORK *between *Medicago *species

**Figure 1:**
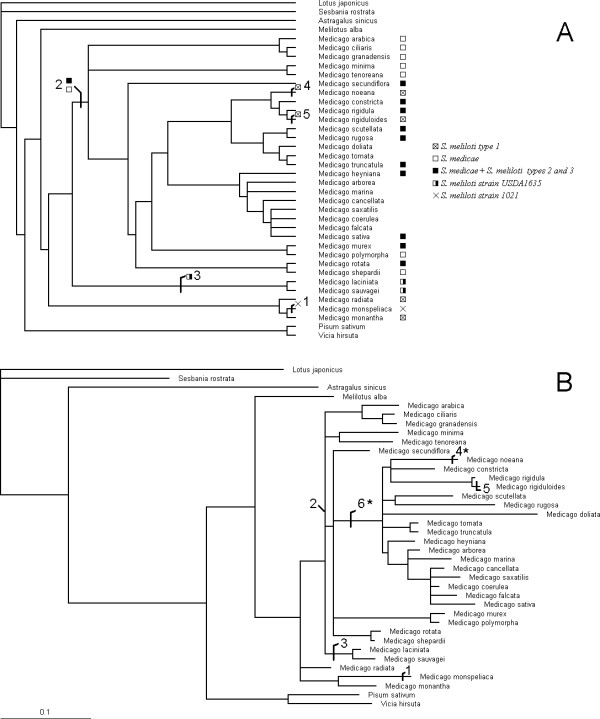
**Phylogenetic tree of *Medicago *species**. A. The topology is from ref. [18]. The pattern of rhizobial specificity in extant species as well as reconstruction of ancestral states is from ref. [13]. The numbered branch labels indicate branch where most likely shifts in rhizobial specificity occurred. B. Phylogram with branch lengths estimated with the codon model (model M8). The numbered branches are those used for testing positive selection (1–5: same as in Figure 1A; 6: noticed for having an unexpected length). Asterisks indicate branches with an *ω *significantly higher than in the rest of the tree. The scale for branch length represents 0.1 nucleotide substitution per codon.

The phylogenetic relationships between *Medicago *species have been examined in a previous study using a nuclear locus [18]. The analyses described below have been performed using the branching order (the topology) found in this study. The unrooted phylogeny we used is displayed in Figure 1A. At first, only between-site variation of selective constraints was considered. We fitted two models of site variation of *ω*, the non-synonymous to synonymous rate ratio (Table 1). The comparison of these models allows detecting positive selection [19]. The model M8A is the null model and doesn't allow positive selection. Variation in the strength of purifying selection among sites is modeled by a Beta distribution controlled by two parameters (*p *and *q*), and an additional category fixed to *ω *= 1 is implemented. In M8 this last *ω *value is allowed to vary beyond one, while all other features are the same. We find that the maximum likelihood estimate of *ω *in M8 is 2.37 (more than twice more non-synonymous than synonymous substitutions, therefore reflecting positive selection). The frequency of these sites under positive selection is 0.10 (close to 38 amino acid sites). There is a large and unambiguously significant gap in likelihood between M8A and M8 (likelihood ratio = 54.44, *P *< 10^-12^), strongly supporting the finding of signatures of positive selection in *NORK*.

**Table 1:** Codon based tests of positive selection assuming site variation of *ω*

	Null model	Positive selection model
Model name [35, 36]	M8A	M8
Transition/transversion ratio	2.17	2.37
Parameter *p *in Beta	3.16	0.96
Parameter *q *in Beta	20.25	3.30
Frequency of additional class	0.19 (*i.e*. 76 sites)	0.10 (*i.e*. 38 sites)
*ω *of additional class (*ω*_s_)	1 (fixed)	2.24
Number of free parameters	4	5
Log-likelihood	-7119.01	-7091.80

Likelihood ratio		54.44
*P*-value		*P *< 10^-12^

### Identification of candidate sites targeted by positive selection

The amount of data at each site of an alignment is considered to be insufficient to allow an independent estimation of *ω *for individual sites. The so-called Bayes empirical Bayes procedure [20] overcomes this problem by using the parameters of the M8 model as a prior distribution of *ω*. The Bayes empirical Bayes procedure allows estimating (i) the probability that a given site belongs to each *ω *category in the model and (ii) a per-site prediction of *ω *along with a confidence interval. Sites displaying a high probability (> 0.95) of belonging to the free *ω *category of model M8 (*ω *= 2.37) are candidate targets of positive selection. Our analysis suggests nine candidate sites with probability > 0.95: 214 (R), 228 (F), 275 (H), 284 (R), 421 (A), 444 (M), 445 (L), 466 (S) and 468 (W). Site numbering and amino acids given are these of *M. truncatula *(GenBank: CAD10809).

**Figure 2:**
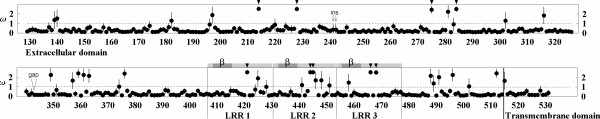
**Prediction of the non-synonymous to synonymous substitution rates ratio (*ω*) for individual sites of *NORK***. Sites are numbered with respect to the reference sequence of *Medicago truncatula *A17 (accession number CAD10809). INS: with respect to this reference sequence, two sites have been inserted between sites 240 and 241 and are not numbered in this figure. GAP: the positions 330 to 342 are missing and the numbering jumps from 329 to 343. The dotted line represents *ω *= 1. Arrowheads: candidate sites for positive selection. Shaded frames: LRRs (solvent-exposed β sheets are marked by darker frames).

Among site variation of *ω *is represented in Figure 2. The nine candidate sites, and more broadly sites with high predicted *ω *values, are scattered in the extracellular domain of NORK (including in the LRRs motifs). The solvent-exposed β sheets of the LRR motifs, which are predicted to control ligand specificity [9], have not undergone positive selection. Interestingly, the C-terminal ends of the three LRRs harbor five of the nine candidate sites, and these sites are at comparable distances from the end of the β sheets. Assuming the spatial structure of other LRR proteins [21], these five candidate sites may be at spatially nearby positions. The four other sites occur in the large N-terminal region of the extracellular domain of NORK which consists of a currently undetermined structural domain.

### Positive selection did not occur preferentially in branches where shifts in rhizobial specificity took place

We tested whether positive selection occurred specifically in the branches where changes in rhizobial preference occurred. We used the test designed by Yang [22] for detecting positive selection in particular branches of a phylogeny. The branches numbered 1–5 correspond to changes in rhizobial specificity (Figure 1A). The branch 6 is selected *a posteriori *because of its unexpected length in the phylogeny of *NORK*, comparing with branch lengths of previous phylogenies of the genus (Figure 1B). This length is defined as nucleotide substitutions per codon and not in non-synonymous substitutions, and therefore there is no *a priori *knowledge of any excess of non-synonymous substitutions in this branch (this is a prerequisite to the application of the test). To our knowledge, branch 6 is not correlated to any known change in phenotype in *Medicago *species. The null model for this test assumes no variation of *ω *between both sites and branches (all branches evolve under a same *ω*_0_). The addition of one additional *ω*_1_, different from the background *ω*_0_, is tested separately in all six chosen branches. For branches 1, 2 and 3, *ω *appears not to be significantly different from other branches (Table 2). Branch 2 is of length 0 (see Figure 1B) and therefore the test is meaningless for this branch. In contrast, *ω *is significantly higher in branches 4 and 6 and significantly smaller in branch 5. It is very unlikely that any non-synonymous substitution occurred in this last branch (*ω *≈ 0). For branches 4 and 6, however, *ω *is not significantly greater than one (Table 2, see Methods).

**Table 2:** Tests of positive selection in specific branches

Branch with free *ω*	*κ*	*ω*_0_	*ω*_1_	np	Log-L	LR	*P*	Test *ω*_1 _> 1
None (null model)	2.32	0.37	None	2	-7298.06			
1 (*M. monspeliaca*)	2.31	0.37	0.36	3	-7298.06	0.00	> 0.50	-
2 (main group)	2.31	0.37	0.40	3	-7298.06	0.00	> 0.50	-
3 (*M. laciniata, sauvagei*)	2.31	0.37	0.38	3	-7298.06	0.00	> 0.50	-
4 (*M. noeana*)	2.32	0.37	1.13	3	-7295.41	5.30*	< 0.05	*P *> 0.50
5 (*M. rigidula*)	2.31	0.36	0.00	3	-7295.91	4.30*	< 0.05	-
6 (group *a posteriori*)	2.32	0.36	1.90	3	-7294.82	6.47*	< 0.05	*P *> 0.10

Note:

*κ*: transition/transversion ratio; np: number of free parameters; Log-L: log-likelihood; LR: likelihood ratio; *: *P *< 0.05; *P*: *P*-value.

Most branches where changes in rhizobial specificity occurred don't display any increase in the rate of non-synonymous substitutions. The only one that shows such increase is the branch leading to *M. noeana *(branch 4). Conversely, at least one other branch (branch 6) displays an even larger increase (*ω*_1 _= 1.90 for branch 6 to be compared to *ω*_1 _= 1.13 for *M. noeana*). Branch 6 is not associated with the pattern of rhizobial specificity in extant species. These results indicate that positive selection in *NORK *is very unlikely to be connected to changes in rhizobial specificity in the *Medicago *genus.

## Discussion

We have examined the pattern of nucleotide divergence in the coding sequence of *NORK *between species of the genus *Medicago*. We found signatures of positive selection suggesting episodes of molecular adaptation of *NORK *in the history of this genus, but with no evidence of correlation with shifts of rhizobial specificity. Below we first examine one methodological issue that might put into question the finding of positive selection, and then discuss its possible interpretations.

### Are our results robust to tree misspecification?

We used a phylogeny of *Medicago *species previously published, and determined using a different locus than the one used for testing positive selection. The phylogeny built from *NORK *sequences (not shown) is indeed severely conflicting with the one we used. Using a tree inconsistent with the data may restrain the validity of the test for positive selection [23]. To confirm our finding of positive selection, we evaluated the robustness of our results to several tree topologies (including the phylogeny reconstructed with amino acid sequences, which by definition tends to minimize the number of non-synonymous changes). Overall we find that the evidence for positive selection is not as strong but still highly significant [see Additional file 2]. The estimated number of candidate sites tends to drop, but the estimated *ω*_s _is still close to 2 and the positions of candidate sites are qualitatively conserved (at last five of the nine candidate sites are conserved, two in the N-terminal region and one in each LRR).

### Positions of candidate sites in the protein

No candidate site has been identified in the solvent-exposed β sheets of the LRRs. This result contrasts with many previous studies of LRR-containing resistance gene families where positive selection mostly targeted these solvent-exposed sites (*e. g*. [24,25]). However, Caicedo and Shaal [26] noted that a majority of sites under positive selection fell outside of solvent-exposed regions in a study of positive selection in a LRR resistance gene of a *Solanum*,. Therefore the operating mode of positive selection in the evolution of LRRs might vary between genes, presumably resulting from different spatial interactions between LRR proteins and their ligand or ligands.

Four of the nine candidate sites are in the N-terminal region of *NORK *which consists of a currently undetermined structural domain. Resistance proteins containing LRRs often include TIR and NBS domains at a similar position. Their role in determining ligand specificity [27] as well as the presence of positively selected sites in these domains [24,25] have been demonstrated. It is therefore possible that the unknown N-terminal domain of NORK has a role in controlling interactions with other proteins. The position of sites where positive selection occurred might be relevant for the inference of the spatial structure of NORK.

### *NORK *does not seem to control partner choice between *Sinorhizobium *strains in *Medicago*

The occurrence of positive selection in *NORK *being demonstrated, we sought for a hypothesis to explain it. The product of this gene is involved in both rhizobial and endomycorrhizal symbioses [8]. Furthermore, *NORK *may have additional roles. It is possible that it intervenes also in the control of root hair growth [28]. In this context it is difficult to infer which selective constraints have driven the numerous amino acid substitutions we detected. A phylogeny of the genus *Medicago *was previously reconstructed using internal and external spacers of the nuclear ribosomal DNA [18]. This phylogeny allowed to identify five putative events of changes in rhizobial specificity within the genus *Medicago *[13].

Here we tested whether amino acid substitutions of *NORK *have played a role in these changes of specificity, by testing whether more substitutions occurred in branches where these changes occurred. The branches corresponding to the association with a large range of *Sinorhizobium meliloti *and *medicae *strains (branch 2), and to specializations to limited ranges of strains (branches 1 and 3) show very small and non-significant variations of *ω*. *M. noeana *and *M. rigiduloides *are closely related (but not sister) species which seem to have both reverted to an ancestral state. However their branches exhibit contrasted pattern of non-synonymous and synonymous evolution. The branch 4 (leading to *M. noeana*) has an *ω *of 1.13 significantly larger than in the rest of the tree but not significantly larger than one. The branch 5 (leading to *M. rigiduloides*) has a significantly lower, virtually null *ω*. The result for branch 4 is ambiguous because it is not possible to positively discriminate between positive selection and relaxed purifying constraints, since *ω *is not significantly greater than one. Furthermore, the statistical properties of branch-specific *ω*, such as sampling errors, as well as their behavior with varying branch length, have been poorly described. Even if positive selection indeed occurred in the branch leading to *M. noeana*, it must have occurred also in other branches not connected to changes in rhizobial specificity. First, because our initial study having detected positive selection did not use this species [12]. Second, because the branch 6 is an even more likely candidate than branch 4 (albeit not significant either). Therefore it is obvious from the results described in Table 2 that there is no general increase in *ω *in branches associated to changes in rhizobial specificity. Evolving preferences between several groups of *Sinorhizobium *are not a good candidate for explaining the high rates of amino acid replacements in *NORK *in *Medicago*.

### Alternative explanations

The exact role of *NORK *in Nod factor signaling is currently unclear [7,10,11], as well as the way it is involved in its other putative functions (mycorrhizal signaling and control of root hair growth). It is not excluded that the NORK protein interacts directly with Nod factors, putative mycorrhizal signaling molecules, or any kind of ligand. It is therefore difficult to formulate any precise hypothesis for explaining positive selection in *NORK*. Below we only enumerate a few general hypotheses.

The hypothesis tested in this article is that *NORK *evolution has been driven by changes of rhizobial specificity (*i.e*. acquisition of a new partner, or co-speciation of partners). This form of evolution can be detected because former states are conserved in unchanged descendants of ancestral species. Another possibility is a coevolutionary arms race between *Medicago *and *Sinorhizobium*, a process where both partners evolve in response to each other's innovations [29]. This may not be easily detectable because the changes don't occur at speciations and the ancestral states do not persist. This suggestion assumes that *NORK *interacts directly with a rhizobial product, and that the symbiosis generates antagonist-like pressures of selection (through conflicts of interest or episodic parasitism).

As a putative receptor for vesicular arbuscular endomycorrhizae (VAM) signaling, *NORK *might as well be under selective pressures caused by the interaction with mycorrhizal fungi. At first glance, VAM are not expected to be able to require molecular adaptation because of a typical absence of specificity (any change would disrupt the interaction). However hidden specificity caused by specific life history [30], and also possible conflicts of interest in VAM, can be taken on consideration as potential sources of selective pressure.

Genes can be recruited as resistance genes while having an initial different function (*e.g*. [31]). As a regulator of common plant symbioses, *NORK *may be used as an entry route for parasites using nodule/VAM development pathway to infect root tissues. In that case, this gene is likely to behave as a resistance gene. There is some clue that the *Lotus japonicus *homologue of *NORK *is required for parasitic infection by root parasites [32] but this result has not been confirmed in *Medicago*.

Eventually, functional studies of protein interactions in which NORKis involved will help identifying the putative selective pressures it undergoes and consequently narrow down these hypotheses.

## Conclusion

We confirm the occurrence of positive selection in the symbiotic gene *NORK*. We show that positive selection was probably not induced by changes in rhizobial specificity during the evolution of the *Medicago *genus. Positions of amino acid sites exhibiting an excess of substitutions indicate that selection did not target solvent-exposed residues of the LRRs, but other sites, inside and outside the LRRs. These results confirm that *NORK *has undergone crucial selective pressures at least in the *Medicago *genus, but raise questions relative to their origin.

## Methods

### Taxa included in the analysis

We used the mRNA sequences of *NORK *orthologs in six non-*Medicago *legume species available in the GenBank database: *Astragalus sinicus*, *Lotus japonicus *(*SYMRK*), *Melilotus alba*, *Pisum sativum*, *Sesbania rotata *and *Vicia hirsuta *(AY946203, AJ430101, AJ498991, AJ438375, AY751547 and AJ428990, respectively). We selected 32 *Medicago *species for sequencing. Some of them can be considered as models: *M. sativa *and its relatives *M. coerulea *and *M. falcata *are important crops. *M. truncatula *is a model in several respects. *M. noeana*, *M. laciniata *and *M. monspeliaca *possess specific rhizobial symbionts that are not associated with other species. We sampled species to represent different symbiotic groups defined on the basis of rhizobial specificity documented in the genus *Medicago *[18]. The material used in this study is listed in Additional file 1, along with the accession numbers of sequences that have been produced.

### Sequencing procedure

One or two genotypes were selected for each *Medicago *species [see Additional file 1]. Total DNA was extracted from 100 mg of frozen leaves according to DNeasy Plant Mini Kit (Qiagen) with the following modification: 1% of Polyvinylpyrrolidone (PVP 40,000) was added to buffer AP1. A region of *NORK *located in the extracellular domain of the protein was amplified and sequenced. Sequence and genic localization of primers are displayed in Additional file 3. For all species, amplification and sequencing were performed as previously reported [12]. For species exhibiting a signal of sequence heterozygosity, sequence data were obtained after cloning. In this case, PCR amplifications were performed using a proofreading DNA polymerase (Expand High Fidelity PCR System, Roche). The PCR products were cloned using the pGEMT-easy cloning kit (Promega) according to the manufacturer protocol. One positive clone per individual was sequenced in both directions with universal SP6 and T7 primers using a fluorescent sequencing system (BigDye Terminator version 3.1 Cycle Sequencing Kit, Applera) and analyzed on an ABI PRISM 3100 semi automated sequencer (Applera). Sequences were assembled and aligned using programs of the Staden package release 1.6.0 beta 4 [33].

### Tests of positive selection

Coding sequences were analyzed with the codeml software from the PAML package version 3.15 [34] under a family of codon substitution models allowing for variation in the non-synonymous to synonymous rate ratio (*ω*) among sites [19] or among branches [22]. We used subsequent modifications of some of these models to ensure adequate hypothesis testing [35,36]. We performed three different tests. The first one compares two models allowing for variation of *ω *between sites but not between branches (M8A and M8) and allows for testing for the presence of sites under positive selection. The second one compares a fixed-*ω *(M1*ω*) model to a two-*ω *(M2*ω*) model where *ω *is allowed to vary in a given pre-defined branch. We used branches 1–5 in Figure 1A which correspond to putative ancestral changes in rhizobial specificity, and branch 6 which was noticed for having an unexpected length. This test only allows verifying that the two *ω *are different. The third test compared any two-*ω *model (where *ω *varies in one given branch) to a version where *ω *in the tested branch is constrained to one (M2*ω*A). This test allowed testing whether *ω *in branches 4 and 6 was significantly greater than one. The statistical tests of M8 vs. M8A, M2*ω *vs. M1*ω *and M2*ω *vs. M1*ω*A were all performed with likelihood ratio tests with one degree of freedom (each couple of models differs by one free parameter).

## Authors' contributions

SDM, TB and JR conceived the study. SS and SDM obtained the sequence data. SDM analyzed the data. SDM, JR and TB discussed the results and wrote the manuscript. All authors read and approved the final manuscript.

## Supplementary Material

Additional File 2 **Parameter estimates and likelihood codon substitution models for alternative tree topologies**. This table presents results of the test of positive selection (allowing for variation between sites), repeated with several alternative tree topologies. Click here for file

Additional File 1 **Species used in this study with their genotype identifier, sample origin and biological information**. This table contains information about biological samples and EMBL/GenBank accession numbers of sequences. Click here for file

Additional File 3 This file contains the sequence of primers used in this study, the localization of primers in the *NORK *gene sequence, and information related to alignment gaps. Click here for file
