# Supplementary material for: Adaptive evolution of the symbiotic gene NORK is not correlated with shifts of rhizobial specificity in the genus Medicago
Source: BMC Evol Biol. 2007 Nov 6;7:210. doi: 10.1186/1471-2148-7-210 (PMC2247475; doi:10.1186/1471-2148-7-210)
Supplement: Additional File 2 — Parameter estimates and likelihood codon substitution models for alternative tree topologies. This table presents results of the test of positive selection (allowing for variation between sites), repeated with several alternative tree topologies. [file 1471-2148-7-210-S2.pdf]

Table S2. Parameter estimates and likelihood codon substitution models for alternative tree topologies.

The topologies are: star topology with no resolved node; topology adapted from Béna *et al.* 2001 (the topology used in this study); topology estimated from coding sequences; topology estimated from protein sequences. The phylogenetic reconstructions were done by maximum likelihood with the software PHYML (HKY model for nucleotide sequences and JTT for amino acid sequences).

The results obtained below were obtained using the same method as in the main text. The null model M8A was compared with the positive selection model M8 for all topologies. Results presented are: log-L: log-likelihood; np: number of parameters estimated (branch lengths + substitution model); tree length (expressed as the expected number of nucleotide substitution per codon); κ: transition/transversion rate ratio; p, q: parameters of the beta distribution; p_1: proportion of sites belonging the supplementary class; ω_s: ω of the supplementary class (fixed to 1 in M8A); sites: sites assigned to the supplementary class (with posterior probability > 0.95; 9 candidate sites presented in the main text are in bold; sites falling in the LRRs are indicated); LRT: likelihood ratio test; P: probability of the LRT.

Topology	Star	Béna *et al.* 2001	Coding sequences	Protein
**M8A**				
log-L	-8934.58	-7119.01	-6666.10	-6693.01
np	38+4	69+4	73+4	73+4
tree length	5.32520	3.16181	2.78345	2.80873
κ	2.03732	2.16509	2.35181	2.35304
p	0.65555	3.16437	2.64667	2.67045
q	5.13798	20.24997	13.71929	13.85478
p_1	0.15914	0.19316	0.19437	0.18446
**M8**				
log-L	-8794.49	-7091.80	-6658.47	-6683.68
np	38+5	69+5	73+5	73+5
tree length	5.73666	3.21809	2.81494	2.84488
κ	2.54808	2.36500	2.43598	2.44200
p	0.37836	0.95656	0.88541	0.89155
q	1.29420	3.29652	2.56778	2.60457
p_1	0.08673	0.09619	0.06896	0.06245
ω_s	3.71313	2.23629	1.97117	2.08797
sites	**214**<br>**228**<br>**275**<br>**284**<br>316<br>359<br>361<br>363<br>374<br>403<br>**421 LRR**<br>425 LRR<br>441 LRR<br>**444 LRR**<br>**445 LRR**<br>458 LRR<br>**466 LRR**<br>**468 LRR**<br>488<br>489<br>491<br>496<br>512	**214**<br>**228**<br>**275**<br>**284**<br>**421 LRR**<br>**444 LRR**<br>**445 LRR**<br>**466 LRR**<br>**468 LRR**	**214**<br>**228**<br>281<br>**421 LRR**<br>**445 LRR**<br>**466 LRR**	**214**<br>**228**<br>**421 LRR**<br>**445 LRR**<br>**466 LRR**
LRT	280.16	54.44	15.27	18.66
P	$<10^{-62}$	$<10^{-12}$	$<10^{-6}$	$<10^{-4}$
