# Supplementary material for: Adaptive evolution of the symbiotic gene NORK is not correlated with shifts of rhizobial specificity in the genus Medicago
Source: BMC Evol Biol. 2007 Nov 6;7:210. doi: 10.1186/1471-2148-7-210 (PMC2247475; doi:10.1186/1471-2148-7-210)
Supplement: Additional File 1 — Species used in this study with their genotype identifier, sample origin and biological information. This table contains information about biological samples and EMBL/GenBank accession numbers of sequences. [file 1471-2148-7-210-S1.pdf]

De Mita *et al.* Adaptive evolution of NORK targets specific sites but is not correlated with shifts of rhizobial specificity.

Table S1. Species used in this study with their genotype identifier, sample origin and biological information.

Species	EMBL accessions	Samples used[1]	Germplasm accession[2]	Life history	Specificity group[3]
M. arabica	AM403530	ES057 (a)		Annual selfer	4
	AM404189	F34022 (b)	L00909		
M. arborea	AM403531<br>AM404190	Melagòn		Perennial outcrosser	not tested
M. cancellata	AM403532<br>AM404191	2731		Perennial outcrosser	n.d.
M. ciliaris	AM403533<br>AM404192	DZA204	L00897	Annual selfer	3
M. coerulea	AJ884582	2839		Perennial outcrosser	not tested
M. constricta	AM403535<br>AM404194	SA24059	L00905	Annual selfer	3
M. doliata	AM403536<br>AM404195	DZA231-D	L00910	Annual selfer	not tested
M. falcata	AM403537<br>AM404196	GR079		Perennial outcrosser	not tested
M. granadensis	AM403538<br>AM404197	2600		Annual selfer	4
M. heyniana	AM403539<br>AM404198	SA12027	L00915	Annual selfer	3
M. laciniata	AM403540<br>AM404199	DZA239	L00904	Annual selfer	2
M. marina	AM403541<br>AM404200	CarnonR96		Perennial selfer	3
M. minima	AM403542<br>AM404201	ES016		Annual selfer	3
M. monantha	AM403543<br>AM404202	PI203475	L00919	Annual selfer	1
M. monspeliaca	AM403544<br>AM404203	Coll.G. Béna	L00920	Annual selfer	5
M. murex	AM403545	SA23199 (a)		Annual selfer	3
	AM404204	PI495338 (b)			
M. noeana	AM403546<br>AM404205	SA2526		Annual selfer	1
M. polymorpha	AM403547<br>AM404206	F34003	L00911	Annual selfer	4
M. radiata	AM403548	118994 (a)		Annual selfer	1
	AM404207	SA28466 (b)	L00893		
M. rigidula	AM403549<br>AM404208	ES024	L00901	Annual selfer	4
M. rigiduloides	AM403550	CI716 (a)		Annual selfer	1
	AM404209	ICISIS (b)	L00899		

continuing on next page

Table S1. Species used in this study with their genotype identifier, sample origin and biological information (cont.)

Species	EMBL accessions	Samples used[1]	Germplasm accession[2]	Life history	Specificity group[3]
M. rotata	AM403551	SA14095-1 (a)	L00912	Annual selfer	3
	AM404210	SeedCo2 (b)	L00906		
M. rugosa	AM403552	GR026 (a)		Annual selfer	4
	AM404211	PI233254 (b)	L00894		
M. sativa	AM403553	PI464774 (a)		Perennial outcrosser	not tested
	AM404212	MH2 (b)			
M. sauvagei	AM403554<br>AM404213	SA11476		Annual selfer	2
M. saxatilis	AM403555<br>AM404214	5898		No information	not tested
M. scutellata	AM403556	ES103 (a)	L00918	Annual selfer	3
	AM404215	GR045 (b)			
M. secundiflora	AM403557	DZA311 (a)		Annual selfer	3
	AM404216	PI537238 (b)	L00917		
M. shepardii	AM403558	SA16402 (a)	L00898	Annual selfer	4
	AM404217	SA16389 (b)			
M. tenoreana	AM403559	SA04639	L00895	Annual selfer	4
M. tornata	AM404218	ES050		Annual selfer	not tested
M. truncatula	AM403561<br>AM404220	A17	L00738	Annual selfer	3
Astragalus sinicus	AY946203				
Lotus japonicus	AJ430101				
Melilotus alba	AJ498991				
Pisum sativum	AJ438375				
Sesbania rostrata	AY751547				
Vicia hirsuta	AJ428990				

[1]: Samples whose number begins by SA are available through SARDI, Australia, these beginning by PI are available through USDA (http://www.ars-grin.gov/) and other are available at UMR Diversité et Adaptation des Plantes Cultivées, INRA, France.

a: samples sequenced with primers 1-2 (region upstream the LRRs, see Additional File 2).

b: samples sequenced with primers 3-9 (LRR region, see Additional File 2).

[2]: Some samples have been made available as fixed material maintained at UMR Diversité et Adaptation des Plantes Cultivées, INRA, France.

[3]: Specificity groups are defined in Béna *et al.* (2005) by criteria of efficient nodules formed upon inoculation with (1) *Sinorhizobium meliloti* type 1; (2) one specific *Sinorhizobium meliloti* type 1 strain; (3) *S. meliloti* type 3 and *S. medicae* strains; (4) *S. medicae* strains; (5) one specific *S. meliloti* type 3 strain.

Reference: Béna G, Lyet A, Huguet T, Olivieri I: **Medicago - Sinorhizobium symbiotic specificity evolution and the geographic expansion of Medicago**. *J. Evol. Biol.* 2005, 18:1547-1558.
