# Supplementary material for: Adaptive evolution of the symbiotic gene NORK is not correlated with shifts of rhizobial specificity in the genus Medicago
Source: BMC Evol Biol. 2007 Nov 6;7:210. doi: 10.1186/1471-2148-7-210 (PMC2247475; doi:10.1186/1471-2148-7-210)
Supplement: Additional File 3 — This file contains the sequence of primers used in this study, the localization of primers in the NORK gene sequence, and information related to alignment gaps. [file 1471-2148-7-210-S3.pdf]

A. sequence of primers (5' – 3')

```
1:    TCGATCGGGGTAACAGAACT
2:    GATCCAGATGCCTTGACTAA
3:    AGTTTGGACCCCTTTTGAA
4:    TGAAGAGACCAACCAAAAAG
5:    GTTGGAGTGGAGACCCTTGT
6:    TCAATTTAACTGTTTATCC
7:    TCTTTCTTCCAATAATCTCA
8:    ATGTGGCAGTGAGATAATGG
9:    TTCCAACAGCCAAAGTAATC
```

B. Localization of primers in *NORK*

The schema below represents the sequence of *NORK* in *Medicago truncatula* (AJ418370)
White and black boxes: exons encoding extracellular domain (black: LRRs)
Light gray box: exon encoding transmembrane domain
Dark gray boxes: exons encoding kinase domain

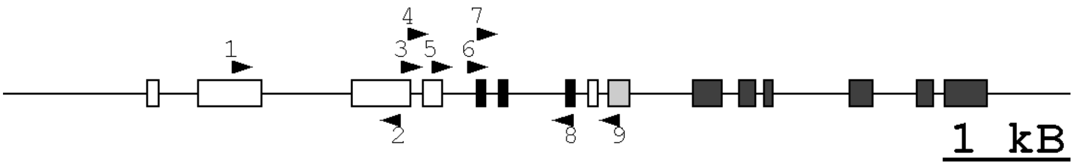

C. Alignment gaps

When the alignment of sequences introduced gaps within coding sequences, it was made sure that all gaps were consistent with the reading frame (in other words, that complete codons were inserted or deleted). The methods we used for analyzing patterns of sequence evolution do not consider insertions and deletions and alignment gaps were considered as missing data in all computations.
